# Effects of continuous straw returning on bacterial community structure and enzyme activities in rape-rice soil aggregates

**DOI:** 10.1038/s41598-023-28747-1

**Published:** 2023-02-09

**Authors:** Luhong Yuan, Yue Gao, Ying Mei, Jiaren Liu, Yusef Kianpoor Kalkhajeh, Hongxiang Hu, Jieying Huang

**Affiliations:** 1 Anhui Province Key Lab of Farmland Ecological Conservation and Pollution Prevention, School of Resource and Environment, Anhui Agricultural University, Hefei, 230036 China; 2 Department of Environmental Science, College of Science and Technology, Wenzhou-Kean University, 88 Daxue Road, Ouhai, Wenzhou, 325060 Zhejiang China

**Keywords:** Biogeochemistry, Environmental sciences

## Abstract

Straw returning is an effective management measure to improve or maintain soil fertility in agricultural ecosystems. This study investigated the effects of straw returning combined with compound fertilizer on the bacterial community, enzyme activities, and soil nutrients’ contents in a rape-rice rotation soil aggregates. To do so, a 5-year field trial (November 2016 to October 2021) was carried out in a paddy soil with three treatments: no straw + no fertilization (CK), compound fertilizer (F), and straw returning + compound fertilizer (SF). Soil aggregates were classified into mega-aggregates (> 2 mm), macro-aggregates (0.25–2 mm), micro-aggregates (0.053–0.25 mm), and silt–clay (< 0.053 mm) using the wet sieve method. High-throughput sequencing was employed to characterize the bacterial community, and Pearson correlation coefficient was used to identify the relationships among bacterial community, organic carbon, nitrogen, phosphorus, and enzyme activities in soil aggregates. Compared with F, the results showed that straw returning increased the content of > 2 mm aggregates by 3.17% and significantly decreased the content of 0.053–0.25 mm aggregates by 20.27%. The contents of organic carbon and total nitrogen in > 0.053 mm straw amended aggregates increased by 15.29 and 18.25%, respectively. Straw returning significantly increased the urease activity of > 0.053 mm aggregates with an average of 43.08%, while it decreased the phosphatase and invertase activities of soil aggregates by 7.71–40.66%. The Shannon indices of the bacterial community in each particle sizes soil aggregates decreased by an average of 1.16% and the Chao indices of the bacterial community in < 2 mm aggregates increased by an average of 3.90% in straw amended soils. Nevertheless, the relative abundances of Chloroflexi and Nitrospirotain in all soil aggregates increased by 6.17–71.77% in straw amended soils. Altogether, our findings suggest that straw returning is an efficient approach to enhance soil structure, carbon and nitrogen contents, and the richness of soil bacterial diversity.

## Introduction

As an important renewable resource, crop straw is not only rich in carbon (C), nitrogen (N), phosphorus (P), potassium (K), and trace elements, but also it contains a large amount of organic components such as lignin and cellulose^1^. Straw returning to the soil significantly improves soil quality indicators, such as the contents of major nutrients, soil structure, and biological functions^2–4^.

Aggregates are among the most important soil physical properties, that play important roles in maintaining the microbial community structure and enzyme activities in soil^5,6^. Soil microbes are the most active biological factors in aggregates; metabolites and enzymes released by their metabolic activities affect the transformation of soil nutrients, material circulations, and the aggregation of soil particles^7^. In turn, the dynamic changes of soil aggregates impact the growth and the activities of soil microbes, the release of microbial enzymes/organic substrates, and the interaction of food webs and the influential microbial turnover^5,8,9^. Soil enzymes have crucial impacts on the transformation of major nutrients^10^. Bacteria is the most abundant soil microorganism, reflecting soil environmental quality/health^11^. Upon straw returning, soil bacterial community and enzyme activities closely contribute to the formation of aggregates^12^. Thus, it is of great importance to study the bacterial community structure and enzymes’ activities of straw amended soil aggregates.

Research has shown that straw returning in wheat-corn rotation significantly increased the microbial abundance of < 0.25 mm soil aggregates, and the bacteria were the dominant microorganism in different particle sizes aggregates^13^. Bai et al.^14^ suggested that straw returning in rice–wheat rotation caused significant changes in the structure of bacterial communities of soil aggregates. Lu et al.^15^ via a 27-year field trial in the dry land farming experimental station, Northern China, found that corn straw returning combined with N and P application increased the enzyme activity of large aggregates, and the soil enzyme activity had positive correlation with the bacterial biomass in > 0.25 mm aggregates. Zhu et al.^16^ showed that a 5-year corn straw returning practice promoted the formation of large soil aggregates, the richness and the diversity of soil bacteria, and the relative abundances of Acidobacteria and Chloroflexi. Liu et al.^17^ found that straw returning combined with chemical fertilizer to a rape-rice rotation increased the activities of invertase and urease in < 0.002 mm aggregates.

It has been well-stablished that farming practices may lead to significant changes in soil physico-chemical, biological, and biochemical properties^18,19^. As such, various crop types/rotations, irrigation rate/frequency, fertilization schemes, and different tillage practices may result in different impacts on soil properties.

Therefore, this study aimed to explore the effects of straw returning on soil aggregates, bacterial communities, enzyme activities, as well as the contents of N, P, C, of aggregates in a rice-rape rotation system. Based upon which, we hypothesized that: (1) straw returning would increase the content/proportion of soil large aggregates; (2) straw returning would reduce the richness and the diversity of bacterial communities of soil aggregates; (3) straw returning would increase the contents of C, N, and P in large aggregates; and (4) soil N, P, K, and enzyme activities would affect the abundance of bacterial communities in soil aggregates.

## Results

### Soil aggregates

Soil aggregates were dominated by mega-aggregates (> 2 mm) and silt–clay fractions (< 0.053 mm), accounting for about 78% (Table 1). Nevertheless, no significant differences appeared in the contents of 0.25–2 and < 0.053 mm aggregates among different treatments. Compared with CK, the content of > 2 mm aggregates increased by 7.25% in F and 10.64% in SF. Unlike, the content of 0.053–0.25 mm aggregates decreased by 17.42% in F and 34.15% in SF compared with CK. The content of > 2 mm SF aggregates increased by 3.17%, and that of 0.053–0.25 mm decreased by 20.27% compared with F. Our results revealed that both F and SF treatments increased the content of mega-aggregates and decreased the content of micro-aggregates. It is also worth mentioning that straw returning combined with compound fertilizer had a greater impact on the composition of soil aggregates, facilitating the conversion of micro-aggregates into the largest ones.

**Table 1:** Contents of soil aggregates in different treatments.

Treatments	Composition of soil aggregate fractions (%)			
	> 2 mm	0.25–2 mm	0.053–0.25 mm	< 0.053 mm
CK	42.51b	8.05a	17.21a	32.23a
F	45.59a	7.70a	14.21b	32.51a
SF	47.03a	7.03a	11.33c	34.61a

Note within columns, means followed by the same letter are not significantly different according to LSD (0.05).

### Distribution of carbon, nitrogen, and phosphorus in soil aggregates

As presented in Table 2, fluctuations took place in the contents of soil major nutrients in relation to the aggregate sizes. Overall, the soil nutrients were higher in macro-aggregates (0.25–2 mm) and micro-aggregates (0.053–0.25 mm) compared with mega-aggregates (> 2 mm) and silt–clay (< 0.053 mm). Except for < 0.053 mm aggregates, SF caused the highest increases in the contents of different nutrients of all soil aggregates. For instance, SF significantly increased the SOC contents of 0.25–2 mm and 0.053–0.25 mm aggregates by 18.16 and 35.36%, respectively, compared with CK, and 22.6 and 20.1%, respectively, compared with F. In all three treatments, however, < 0.053 mm soil aggregates had the significantly lowest SOC, TP, and TN contents.

**Table 2:** Effects of different treatments on SOC, TN, and TP of soil aggregates.

Soil variables	Treatments	Aggregate size			
		> 2 mm	0.25–2 mm	0.053–0.25 mm	< 0.053 mm
SOC (g kg^−1^)	CK	18.42a	28.03b	24.94c	14.68a
	F	19.33a	27.03b	28.11b	14.38a
	SF	19.95a	33.12a	33.75a	13.79a
TN (g kg^−1^)	CK	1.70a	2.42a	2.19a	1.56a
	F	1.77a	2.26a	2.60a	1.51a
	SF	1.86a	2.93a	3.13a	1.50a
TP (g kg^−1^)	CK	0.56b	0.70a	0.78a	0.55a
	F	0.83ab	0.77a	0.67a	0.49a
	SF	0.87a	0.80a	0.90a	0.61a

Note within columns, means followed by the same letter are not significantly different according to LSD (0.05).

### Enzyme activities in soil aggregates

**Figure 1:**
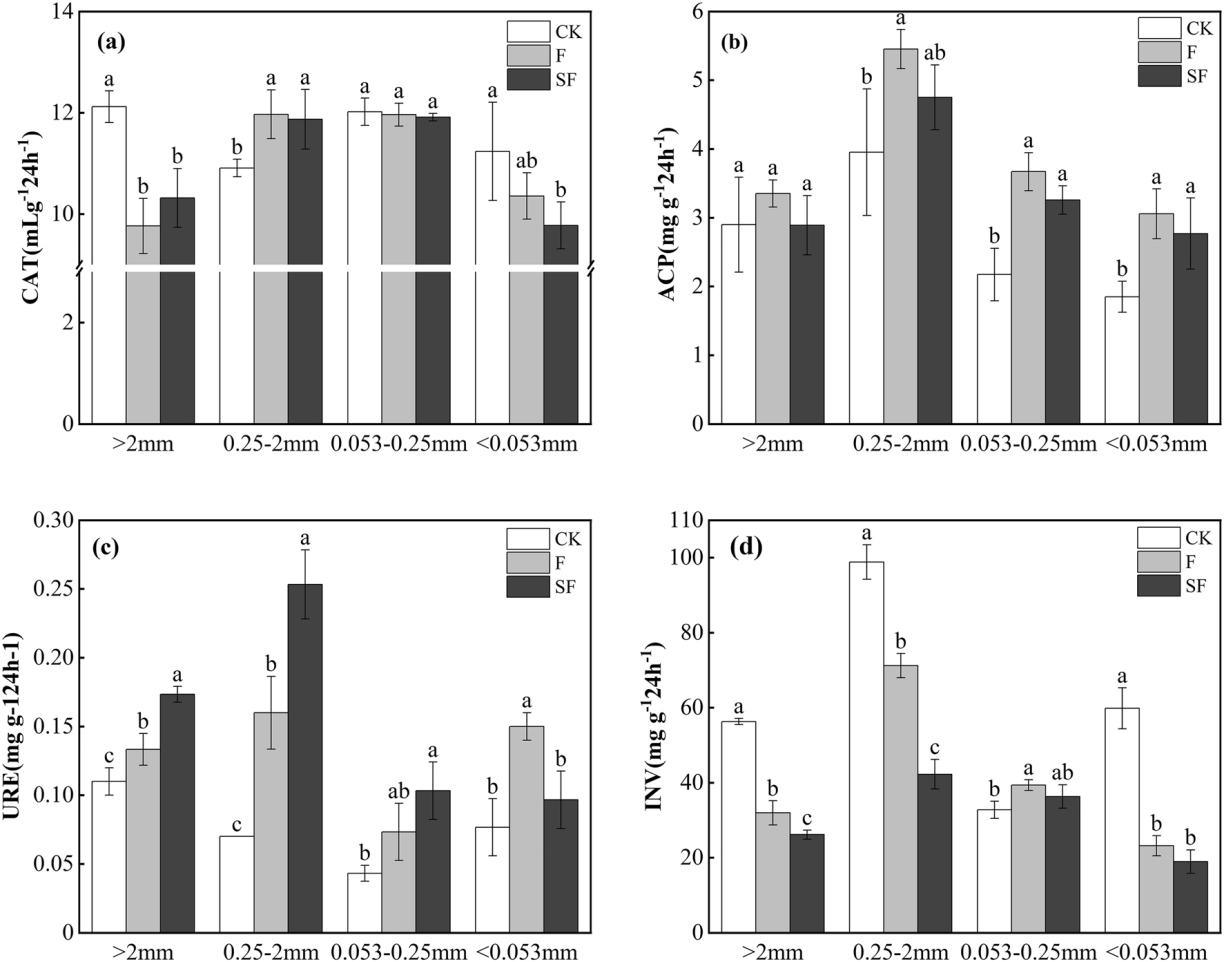
Enzyme activity in soil aggregates under different treatments. They are catalase (CAT), acid phosphatase (ACP), urease (URE), and invertase (INV). The error bars represent the standard errors. The letter in the figure represents the significant differences according to LSD (0.05).

Both F and SF treatments reduced CAT and INV in > 2 mm and < 0.053 mm aggregates compared with CK (Fig. 1). SF increased CAT in > 2 mm aggregates compared with F treatment. Compared with CK, CAT was significantly reduced in > 2 mm SF and F aggregates by 19.42 and 14.85%, respectively, while increases happened in 0.25–2 mm aggregates by 8.80 and 9.72%, respectively (Fig. 1a). Compared with F, SF increased and decreased CAT in > 2 mm and < 0.053 mm aggregates, respectively, by 5.67 and 5.6%, respectively. Compared with CK, INV of all F soil aggregates decreased by 27.98–61.28%, while that of SF decreased by 53.55–68.30% (Fig. 1d). Also, SF reduced INV in all soil aggregates by 7.71–40.66% compared with F.

Unlike, both F and SF treatments increased ACP and URE in all soil aggregates compared with CK (Fig. 1). Accordingly, F increased ACP of all soil aggregates by 15.63–68.87%, while SF increased ACP of 0.25–2 mm to < 0.053 mm aggregates by 20.15–49.85% (Fig. 1b). Compared with F, SF decreased ACP in all soil aggregates by 9.38–13.82%. Compared with CK, F and SF increased URE of all soil aggregates by 21.18–128.57% and 26.08–261.86%, respectively, (Fig. 1c). Compared with F, SF increased and decreased URE in > 2 mm to 0.053–0.25 mm and < 0.053 mm aggregates, respectively, by 30–58.3% and 35.5%, respectively.

### Bacterial diversity of soil aggregates

The diversities of bacterial alpha of aggregates under different treatments are summarized in Table 3. Compared with CK, F increased and decreased the Chao indices in > 2 mm and 0.25–2 mm to < 0.053 mm aggregates, respectively, by 2.71 and 1.73–9.53%, respectively; SF decreased the Chao index in > 2 mm to < 0.053 mm aggregates by 0.51–4.36%; F increased the Shannon index of > 2 mm aggregates by 2.3%; SF decreased the Shannon index of 0.053–0.25 mm aggregates by 1.25%. Compared with F, SF decreased and increased the Chao indices of > 2 mm and 0.25–2 mm to < 0.053 mm aggregates, respectively, by 5.60 and 1.25%%–5.71%, respectively; SF significantly decreased the Shannon indices of > 2 mm to 0.053–0.25 mm aggregates by 0.42–2.93%. In conclusion, straw returning reduced the richness and the diversity of the bacterial community.

**Table 3:** Analysis of soil bacterial community alpha diversity at soil aggregates.

Diversity index	Treatments	Aggregate size			
		> 2 mm	0.25–2 mm	0.053–0.25 mm	< 0.053 mm
Chao	CK	5018.74b	6266.74a	6008.35a	6033.56a
	F	5154.94a	5754.40c	5904.34c	5458.59c
	SF	4866.33c	6027.06b	5977.90b	5770.28b
Shannon	CK	10.34b	10.96a	10.77a	10.79a
	F	10.58a	10.93b	10.77a	10.68b
	SF	10.27c	10.88c	10.64b	10.68b

Note within columns, means followed by the same letter are not significantly different according to LSD (0.05).

### Bacterial community structure in soil aggregates

**Figure 2:**
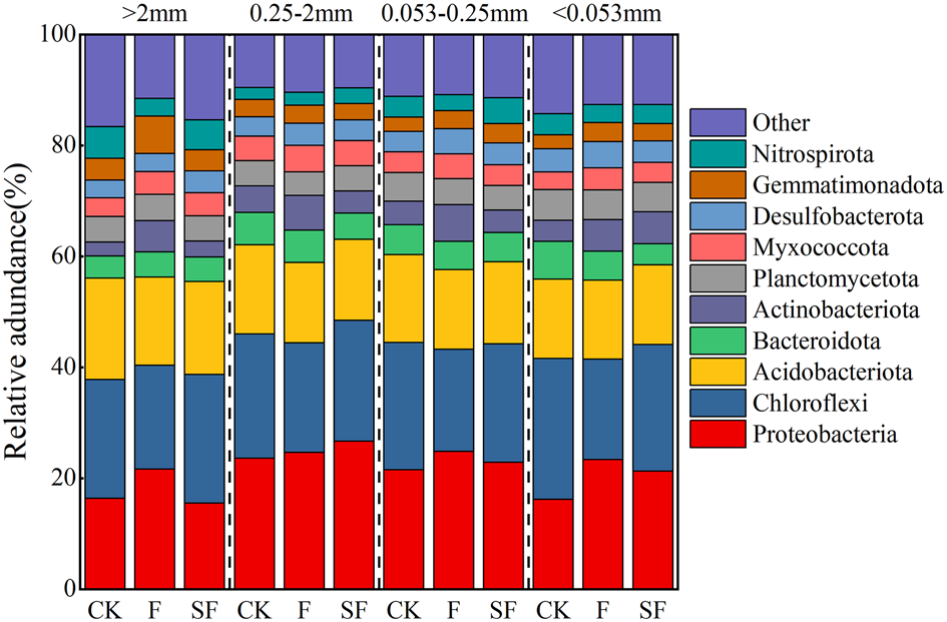
Relative abundance of phylum level for bacterial communities in different soil aggregates. The category “other” includes unclassified microbes and other less abundant taxa.

Bacterial communities of soil aggregates at the phylum level were ranked by the abundance as Proteobacteria, Chloroflexi, Acidobacteriota, Bacteroidota, Actinobacteriota, Planctomycetota, Myxococcota, Desulfobacterota, Gemmatimonadota, Nitrospirota, etc. The top ten categories accounted for 83.4–90.4% of the total bacteria, with the dominant bacterial phylum (abundance > 7%). In all soil aggregates, Proteobacteria had the highest abundance (15.5–26.7%) and Nitrospirota had the lowest abundance (2.16–5.72%) (Fig. 2). Compared with CK, F increased the relative abundances of Proteobacteria, Actinobacteriota, Myxococcota, Desulfobacterota, and Gemmatimonadota in all soil aggregates by 1.30–127% and those of Bacteroidota and Planctomycetota in > 2 mm aggregates by 14.4 and 1.47%, respectively. Unlike, F, compared with CK, decreased the relative abundances of Chloroflexi, Acidobacteriota, and Nitrospirota in all soil aggregates by 0.72–44.8% and those of Bacteroidota and Planctomycetota in 0.053–0.25 mm to < 0.053 mm aggregates by 3.04–23.8%. Compared with CK, SF increased the relative abundances of Chloroflexi, Bacteroidota, Actinobacteriota, Myxococcota, and Desulfobacterota in > 2 mm aggregates by 8.37–25% and those of Proteobacteria, Nitrospirota, and Desulfobacterota in 0.25–2 mm to 0.053–0.25 mm aggregates by 6.33–29.2%. Herein, the relative abundances of Chloroflexi, Bacteroidota, and Planctomycetota decreased in 0.25–2 mm to < 0.053 mm aggregates by 0.75–44.7%. Compared with F, SF increased the relative abundances of Chloroflexi, Acidobacteriota, and Nitrospirota in all soil aggregates by 0.99–71.8% and those of Proteobacteria and Planctomycetota in 0.25–2 mm aggregates by 8.22 and 6.94% respectively. On contrary, SF decreased the relative abundances of Bacteroidota, Actinobacteriota, and Gemmatimonadota in > 2 mm to 0.25–2 mm aggregates by 3.23–49% and those of Proteobacteria, Planctomycetota, Myxococcota, and Desulfobacterota in 0.053–0.25 mm to < 0.053 mm aggregates by 1.6–38.9%.

### Linkages among the dominant bacterial phylum, soil major nutrients, and enzyme activities of soil aggregates

**Figure 3:**
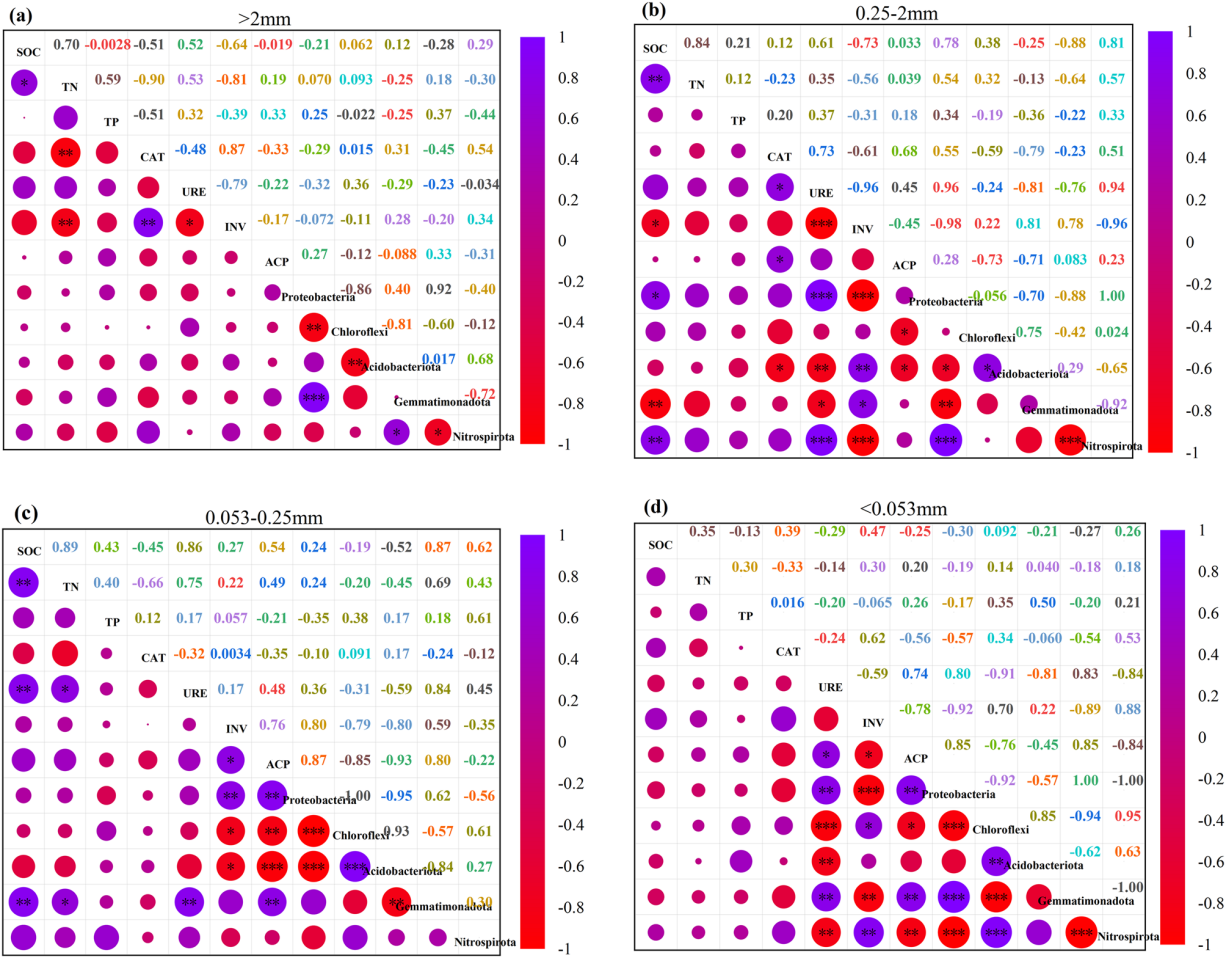
Correlation analysis of bacterial communities, SOC, TN, TP, and enzyme activity in soil aggregates. *Significant at the 0.05 probability level. **Significant at the 0.01 probability level. ***Significant at the 0.001 probability level.

Pearson correlation analysis was performed to discern the linkages among the dominant bacterial communities with SOC, TN, TP, and enzyme activities (Fig. 3). Our results revealed insignificant correlations among the dominant bacterial population, enzyme activities, SOC, TN, and TP in > 2 mm aggregates (Fig. 3a). In 0.25–2 mm aggregates, Proteobacteria and Nitrospirota had positive correlations with SOC and URE, and negative correlations with INV (Fig. 3b). Herein, Gemmatimonadota had a positive correlation with INV, and negative correlations with SOC and URE; Chloroflexi had a negative correlation with ACP; Acidobacteriota had a positive correlation with INV, and negative correlations with URE, CAT, and ACP. In 0.053–0.25 mm aggregates, ACP and INV had negative correlations with Chloroflexi and Acidobacteriota; Proteobacteria had positive correlations with ACP and INV; Gemmatimonadota had positive correlations with SOC, TN, URE, and ACP; and the correlations between Nitrospirota and enzymes activities, and SOC, TP, and TN were all insignificant (Fig. 3c). In < 0.053 mm aggregates, Proteobacteria and Gemmatimonadota had significant positive correlations with URE and ACP, and a negative correlation with INV; Chloroflexi and Nitrospirota had positive correlations with INV, and negative correlations with URE and ACP; Acidobacteriota had a negative correlation with URE; insignificant correlations occurred among the dominant bacterial populations with SOC, TN, and TP (Fig. 3d). It is worth mentioning that ACP, URE, and INV were the main factors affecting the dominant bacterial community of soil aggregates, followed by CAT and SOC.

## Discussion

Our observations regarding the increases in the proportions of mega-aggregates (> 2 mm) and silt–clay (< 0.053 mm) via both compound fertilization alone and straw returning combined with compound fertilization are in line with the results of Liu et al.^20^. In addition, our findings with respect to the significant reduction of micro-aggregates (0.053–0.25 mm) via straw returning combined with conventional fertilization are consistent with the those of Li et al.^21^ who studied the impact of long-term straw returning on the distribution of paddy soil aggregates in Deyang, Sichuan Province, West China. Straw returning has shown to positively improve soil structure by increasing the aggregation of micro-aggregates/small particles into the larger ones, thus increasing the content and the stability of large aggregates^22^. Crop straw decomposition produces proteins, cellulose, and lignin that, in turn, serve as the core for the formation of large aggregates and adhere to the small aggregates or micro particles through adsorption^23^. Furthermore, fresh straw produces a variety of organic cementing substances during decomposition, enhancing the activity of soil microorganisms/microbial metabolism, thereby promoting the formation of large aggregates^24^. On the other hand, frequent rape-rice rotation causes the breakage of > 0.25 mm aggregates^25^. Accordingly, our results showed the dominance of silt–clay aggregates.

We also found increases in SOC with decreasing the size of aggregates from > 2 mm to 0.053–0.025 mm. Similar findings were reported by Li et al.^26^. Increases in SOC content of aggregates with the decreasing the particle size might attribute to the larger specific surface area and the higher cohesive force of the smaller particles^27^. In addition, the SOC content of micro-aggregates mainly consists of stable humus carbon that is continuously accumulated^28^. Compared with F, straw returning combined with compound fertilizer significantly increased the SOC content of > 0.053 mm aggregates which might be due to the stimulation of soil microbial activity and the increase of microbial biomass caused by the continuous addition of straw as exogenous C^29^. The trend of TN accumulation in soil aggregates was consistent with that of SOC. Yan et al.^30^ revealed that small-sized aggregates have a strong ability to preserve N. The adsorption capacity of NH_4_^+^ decreases with increasing the size of aggregates, resulting in the highest TN content of micro-aggregates. Further, straw returning can improve the N fixation capacity of soil aggregates^27^. The released N after the decomposition of straw is easily absorbed and utilized by soil microorganisms, converting the highly mobile inorganic N into the relatively stable organic N^31^. However, due to the short microbial life cycle, the latter is easily adsorbed by soil aggregates of different particle sizes after decomposition, increasing the TN content of soil aggregates. The distribution of TP content in different aggregate classes were rather uniform, which is similar to the results of Shi et al.^32^. This might attribute to the P adsorption/precipitation in soils, causing no significant changes in aggregates’ P^26^.

Results of this study demonstrated that both straw returning combined with compound fertilization and compound fertilization alone significantly increased acid phosphatase and urease activities, and decreased catalase and invertase activities in various soil aggregates. Catalase and invertase are among the main drivers of the decomposition of soil organic matter; acid phosphatase promotes the conversion of soil C, N, and P; and urease specifically contributes to the conversion of soil N^33^. Straw returning provides abundant energy for soil microorganisms, optimizing their living environment, promoting microbial activities, and stimulating the secretion of soil enzymes involved in C, N, and P cycles^34^. Upon the reduction of soil soluble nutrients via changing the conversion rates of organic nutrients, decreases occur in soil enzyme activity^35^. Our results revealed that catalase, acid phosphatase, and invertase were mainly distributed in 0.25–2 mm aggregates^36^, while they had less distribution in < 0.053 mm aggregates. Furthermore, urease had the lowest activity in 0.053–0.25 mm aggregates in all treatments. In Li et al.^37^, the maximum values of invertase and acid phosphatase were found in 1–2 mm aggregates, while that of urease was found in < 0.053 mm aggregates. Liu et al.^38^ found the lowest activities of catalase, invertase, acid phosphatase, and urease in < 0.053 mm aggregates. These results suggest that the level of the aggregates’ stability in various soil types determines the activity of soil enzymes. Besides, the different main substances that bind the aggregates to the various particles may also lead to different binding modes and adsorption capacities of soil enzymes and aggregates^39^.

Soil bacterial diversity is crucial for maintaining soil health, and it changes the patterns of nutrients and other soil attributes following straw returning^40^. In this experiment, the results of 16S rDNA high-throughput sequencing analysis revealed a general reduction in both Chao and Shannon indices of bacteria in SF treated soil aggregates of different sizes. Diversity index is an important index to evaluate the community diversity. Accordingly, higher index indicates higher abundance and homogeneity of bacterial community and more complex community structure. Luan et al.^41^ found that conventional fertilization had no significant effect on bacterial diversity, while maize straw application significantly increased soil bacterial diversity in dryland red soil in Yingtan, East China. Yu et al.^42^ found that deep straw application reduced bacterial richness and evenness. These results suggest that the effect of straw on soil bacteria widely varies among the different treatments and soil environmental conditions. In addition, the anaerobic flooded environment in rice season inhibited the microbial respiration, reducing the microbial biomass and activity and limiting the decomposition and consumption of organic matter^43^. The addition of straw increased the burden of microbial decomposition, thus reducing the bacterial diversity.

In this study, we found that straw returning changed the structural composition of bacterial communities in soil aggregates, such as the relative abundances of phyla Proteobacteria, Gemmatimonadota, and Chloroflexi, all of which changed significantly. Similar observations occurred after a long-term ditch-buried straw returning in a rice–wheat rotation system^44^. Despite providing resources for microbial growth, long-term straw returning changes the corresponding microbial populations^45^. This experiment showed that straw repatriation increased the relative abundance of all Proteobacteria in < 2 mm aggregates, while it decreased Chloroflexi. Proteobacteria belongs to eutrophic bacteria, which can grow rapidly in nutrient rich environments^46^. Chloroflexi belongs to phototrophic bacteria with an anti-stress strategy in nutrient deficiency environments^47^. Further analysis revealed that the relative abundance of Acidobacteriota was decreased in all soil aggregates of different sizes, and those of Gemmatimonadota and Nitrospirota increased significantly in micro-aggregates due to the weak cellulose degrading capability of Acidobacteria^48^. Gemmatimonadota can use straw organic carbon to provide energy for its metabolism and growth^49^. Nitrospirota can convert nitrite to nitrate^50^, becoming a nitrogenous nutrient that can be easily up-taken by the plants and other microbes.

## Conclusion

This study underlines the impacts of in situ co-application of straw and compound fertilizer on bacterial community structure and enzymes activities of rape-rice soil aggregates in Chaohu experimental site, Anhui Province, East China. Our results showed the dominance of > 2 mm and < 0.053 mm aggregates. Compared with F, increase and decrease occurred in the proportions of > 2 and 053–0.25 mm soil aggregates in SF treated soils, suggesting that straw returning facilitates the conversion of small aggregates to the largest ones. We found no significant change in the contents of N and P of soil aggregates in all treatments, although a significant increase took place in SOC of SF treated > 0.053 mm aggregates compared with F. Similarly, increases happened in the contents of URE and ACP of both F and SF treated aggregates compared with the CK. Unlike, both F and SF treatments decreased the contents of INV and CAT in mega-aggregates and silt–clay. SF reduced the diversity of the bacterial community, but it increased the relative abundances of Chloroflexi, Acidobacteriota, and Nitrospirota in soil aggregates compared with F. Proteobacteria, Chloroflexi, Acidobacteriota, Gemmatimonadota, and Nitrospirota were the dominant flora of bacteria in soil aggregates, which had significant relationships with ACP, INV, and URE.

## Methods and materials

### Site description and experimental design

The experimental site was located in Chaohu, Anhui Province, East China (31°39′57″N, 117°40′48″E), with a subtropical humid monsoon climate and a mean annual temperature and precipitation of 18 °C and 1150 mm, respectively. The soil type is gleyed paddy soil. Rape-rice rotation has been established in this station since 2016. This experiment randomly implemented three treatments including: no straw + no fertilizer (control, CK), compound fertilizer (F), straw returning + compound fertilizer (SF). All treatments were replicated three times in 30 m^2^ plots. We applied compound fertilizer (N-P-K: 18-10-18) and urea (N: 46.4%). The fertilization method was in accordance with that practiced by the local farmers. Seasonal fertilization: 524.67 kg ha^−1^ compound fertilizer (N-P-K: 18-10-18) to the rice season as the base fertilizer, 105 kg ha^−1^ urea (N: 46.4%) each time for two topdressings; 502.3 kg ha^−1^ compound fertilizer (N-P-K: 18-10-18) to the rape season as the base fertilizer, and 150 kg ha^−1^ urea (N: 46.4%) each time for two topdressings. After the seasonal harvest of rice/rape crops, we cut the straws by a harvester (with an average length of about 5–8 cm) and ploughed them into the soil. Prior to the experiment, the topsoil (0–20 cm) had a pH of 6.03, TN of 1.47 g kg^−1^, TP of 0.45 g kg^−1^, available P of 12.87 mg kg^−1^, available K of 109.45 mg kg^−1^, and total organic C of 14.09 g kg^−1^.

### Soil sampling and particle-size fractionation

At each plot, topsoil (0–20 cm) samples were collected composed of five sub samples in September 2021. Fresh soils were gently crushed and passed through an 8 mm sieve. Soil aggregates were fractionated using a wet sieving method^51^. A series of sieves were used for aggregate-size fractions: (i) > 2 mm (mega-aggregates); (ii) 0.25–2 mm (macro-aggregates); (iii) 0.053–0.25 mm (micro-aggregates); (iv) < 0.053 mm (silt–clay). Briefly, fresh soil was submerged in sterile water for 10 min. Then, soils were sieved by moving the sieve up and down 30 times/min (approximately 4 cm amplitude) over 5 min under a slight angle to ensure that water and small particles were able to pass through the sieve. The separated aggregates were used for various chemical and molecular analyses. The samples for DNA extraction were stored at – 80 °C. Samples for soil enzyme activity analysis were stored at 4 °C. Samples used for soil carbon, nitrogen, and phosphorus analyses were air dried and stored at room temperature.

### Measurements of soil major nutrients and enzymes’ activities

See Bao^52^ for the determinations of the contents of carbon, nitrogen, and phosphorus in soil. Correspondingly, SOC was oxidized by potassium dichromate; soil TN was determined by the Kjeldahl method; and soil TP was measured with sodium hydroxide-molybdenum antimony colorimetric method. For the details of the determination of soil enzyme activity, please see Guan^53^. Briefly, soil invertase activity (INV) was detected using 3.5-dinitrosalicylic acid colorimetry, and indicated as the number of milligrams of glucose in 1 g dry soil at 37 °C for 24 h. Soil urease activity (URE) was detected using sodium phenol-sodium hypochlorite colorimetry, and indicated as the number of milligrams of NH_3_-N in 1 g dry soil at 37 °C for 24 h. Soil catalase activity (CAT) was detected using potassium permanganate volumetric and indicated as the number of milliliters of 0.02 mol L^−1^ potassium permanganate consumed in 1 g dry soil at 20 °C for 24 h. Soil acid phosphatase activity (ACP) was detected using sodium phenylphosphate colorimetry, and indicated as the number of milligrams of phenol released in 1 g dry soil at 37 °C for 24 h.

### Soil DNA extraction and high-throughput sequencing

The genomic DNA was extracted from 0.5 g of soil aggregates using E.Z.N.A. Soil DNA Isolation Kit (Omega Bio-Tek, Norcross, GA, U.S.), and purified according to the manufacturer’s instructions. The bacterial V4-V5 region was amplified using the primers 515F (5′-GTGCCAGCMGCCGCGG-3′) and 907R (5′-CCGTCAATTCMTTTRAGTTT-3′)^54^. The 16S rRNA gene fragments were sequenced using the MiSeq platform. Amplicons were extracted from 2% agarosegels and purified using the AxyPrep DNA Gel Recovery Kit (AXYGEN) according to the manufacturer’s instructions and were quantified with QuantiFluor -ST (Promega). Purified amplicons were pooled in an equimolar manner, and were then paired end-sequenced on a MiSeq platform. MiSeq sequencing was performed at Biozeron (Shanghai, China).

### Statistical analysis

All comparative analyses were based on the normalized OTU abundance. Comparative analyses of soil physicochemical parameters and bacterial community structure among the treatments were performed using a one-way ANOVA with the LSD test; results with *p* < 0.05 were considered statistically significant (IBM SPPS 21.0, Chicago, IL, USA). The Chao and Shannon indices were calculated using Mothur. Figures were produced using ORIGIN 2021 (Originlab Corporation, Northampton, MA, USA).

### Ethical standards

This project and the experiments were conducted in strict compliance with the IUCN Policy Statement on Research Involving Species at Risk of Extinction and the Convention on the Trade in Endangered Species of Wild Fauna and Flora. This research study was carried out in accordance with the national, international or institutional guidelines.

## Data Availability

The datasets generated and analyzed during the current study are available in the European Nucleotide Archive repository, PRJEB55710.
